# Correction: Mood symptoms predict COVID-19 pandemic distress but not vice versa: An 18-month longitudinal study

**DOI:** 10.1371/journal.pone.0307848

**Published:** 2024-07-22

**Authors:** Benjamin A. Katz, Iftah Yovel

In Fig 2, the red rectangle is missing. Please see the correct Fig 2 here.

**Fig 2:**
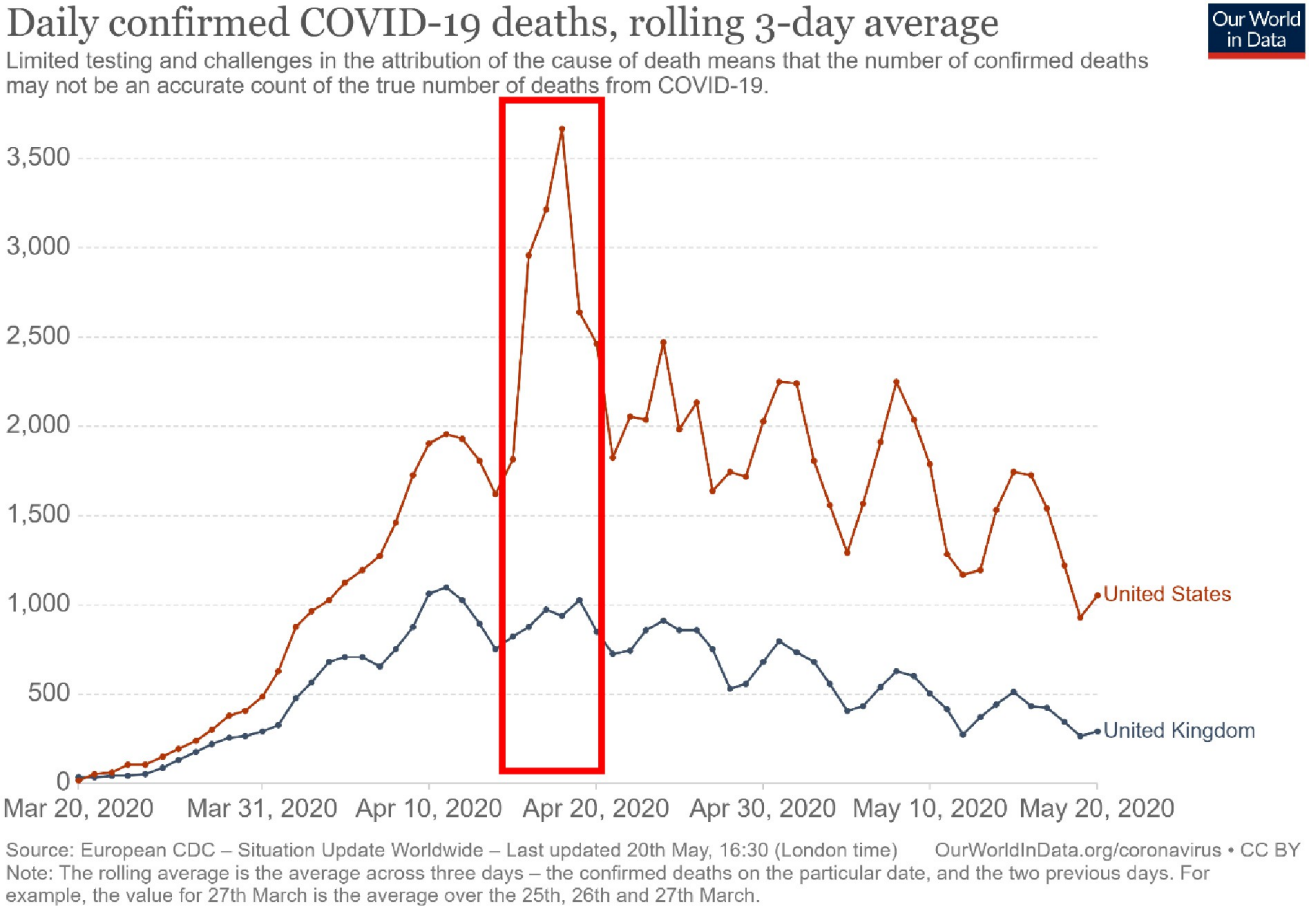
Daily confirmed deaths of COVID-19 between March 20, 2020 and May 20, 2020. Figure retrieved on September 26, 2021 from OurWorldInData.org/coronavirus, which visualized data reported by the European Center for Disease Control. The red rectangle indicates the dates in which the third assessment took place, between April 15 and April 20.
