# Supplementary material for: Mood symptoms predict COVID-19 pandemic distress but not vice versa: An 18-month longitudinal study
Source: PLoS One. 2022 Sep 2;17(9):e0273945. doi: 10.1371/journal.pone.0273945 (PMC9439223; doi:10.1371/journal.pone.0273945)
Supplement: S1 Table — (DOCX) [file pone.0273945.s001.docx]

Supplementary Table 1. Additional demographic information of sample

| Factor | N (% from full sample) |
| --- | --- |
| Gender  Male  Female  Other/Prefer not to say | 97 (44.50%)  118 (54.13%)  3 (1.38%) |
| Current Location  United States  United Kingdom  Canada  Ireland | 102 (46.79%)  100 (45.87%)  33 (15.14%)  1 (0.46%) |
| Race/Ethnicity  White  Hispanic or Latino  Black/Black British/African  American  Mixed/Multiple  Native American/American  Indian  Other/Prefer not to say | 182 (83.49%)  5 (2.29%)  9 (4.13%)  5 (2.29%)  1 (0.46%)  10 (4.59%) |
|  |  |
|  |  |
| Age |  |
| 19-29 | 32 (14.68%) |
| 30-39 | 67 (30.73%) |
| 40-49 | 43 (19.72%) |
| 50-59 | 37 (16.97%) |
| 60+ | 29 (13.30%) |
| Education  Less than 9th grade  9th to 12th grade  High school graduate  Some college (no degree)  Associate's degree  Bachelor's degree  Master's degree  Professional degree  Doctoral degree  Prefer not to say | 1 (0.46%)  4 (1.83%)  31 (14.22%)  45 (20.64%)  9 (4.13%)  84 (38.53%)  30 (13.76%)  5 (2.29%)  7 (3.21%)  2 (0.92%) |
| Employment Status  Employment for wages  Self-employed  Out of work and looking for work  Out of work and not currently looking for work  Homemaker  Student  Retired  Unable to work  Other/Prefer not to say | 104 (47.71%)  43 (19.72%)  9 (4.13%)  8 (3.67%)  13 (5.96%)  9 (4.13%)  16 (7.34%)  13 (5.96%)  3 (1.38%) |
