# Supplementary material for: Mood symptoms predict COVID-19 pandemic distress but not vice versa: An 18-month longitudinal study
Source: PLoS One. 2022 Sep 2;17(9):e0273945. doi: 10.1371/journal.pone.0273945 (PMC9439223; doi:10.1371/journal.pone.0273945)
Supplement: S2 Table — T1 returners are those who returned for T2. T1 dropouts are those who did not return for T2. T2 returners are those who completed T1, T2, and T3. T2 returners are those who completed T1 and T2 but not T3. (DOCX) [file pone.0273945.s002.docx]

**S2 Table. Comparison of participants that did and did not return for later assessments**. T1 returners are those who returned for T2. T1 dropouts are those who did not return for T2. T2 returners are those who completed T1, T2, and T3. T2 returners are those who completed T1 and T2 but not T3..

|  | M (SD) at T1 | | T1-T2 comparison | | |  | M (SD) at T2 | | T2-T3 comparison | | |
| --- | --- | --- | --- | --- | --- | --- | --- | --- | --- | --- | --- |
| Measure | Returned at T2 | Did not return at T2 | *t* | *p* | *d* |  | Returned at T3 | Did not return at T3 | *t* | *p* | *d* |
| Age | 38.48 (12.67) | 34.72 (11.16) | -3.41 | 0.001 | -0.31 |  | 41.90 (13.07) | 34.07 (10.28) | -6.19 | < .001 | -0.65 |
| Gender | 0.43 (0.50) | 0.45 (0.50) | 0.35 | 0.728 | 0.03 |  | 0.46 (0.50) | 0.39 (0.49) | -1.16 | 0.248 | -0.13 |
| Depression | 6.49 (5.57) | 5.66 (5.03) | -1.69 | 0.093 | -0.15 |  | 5.85 (5.64) | 7.76 (5.97) | 2.94 | 0.004 | 0.33 |
| Anxiety | 3.65 (4.12) | 3.33 (3.62) | -0.9 | 0.369 | -0.08 |  | 3.25 (3.91) | 4.33 (4.25) | 2.37 | 0.019 | 0.27 |
