# Supplementary material for: Mood symptoms predict COVID-19 pandemic distress but not vice versa: An 18-month longitudinal study
Source: PLoS One. 2022 Sep 2;17(9):e0273945. doi: 10.1371/journal.pone.0273945 (PMC9439223; doi:10.1371/journal.pone.0273945)

Supplementary File 1. Analyses replicated with only participants from the United States and United Kingdom (N = 202)

Table 1. Stressors related to COVID-19 and cross-sectional correlation with depression and anxiety

| **Variable** | **Mean (SD) /**  **Number in sample (Percent)** | **Depression T3**  **(*r*)** | **Anxiety T3**  **(*r*)** |
| --- | --- | --- | --- |
| **Anxiety/stress as a result of COVID-19 pandemic** | 57.54 (27.51) | .36** | .30** |
| **Loneliness as a result of COVID-19 pandemic** | 42.07 (32.52) | .48** | .32** |
| **Became ill from possible exposure to COVID-19** |  |  |  |
| **Me** | 7 (3.5%) | .16* | .15* |
| **Close to me** | 38 (18.8%) | .11 | .03 |
| **n/a** | 163 (80.6%) | -.09 | -.06 |
| **Knows someone who died from COVID-19** |  |  |  |
| **Me** | 9 (4.5%) | -.02 | .01 |
| **Close to me** | 17 (8.4%) | -.03 | -.03 |
| **n/a** | 179 (88.6%) | -.06 | -.03 |
| **Job requires possible exposure to COVID-19** |  |  |  |
| **Me** | 32 (15.8%) | .05 | .06 |
| **Close to me** | 54 (26.7%) | .04 | .01 |
| **n/a** | 136 (67.3%) | -.03 | -.09 |
| **Lost job or reduced income due to COVID-19 pandemic** |  |  |  |
| **Me** | 56 (27.7%) | .03 | -.01 |
| **Close to me** | 67 (33.2%) | .08 | -.00 |
| **n/a** | 111 (55.0%) | -.10 | -.06 |
| **Increased responsibilities at home due to COVID-19 pandemic** |  |  |  |
| **Me** | 66 (32.6%) | .03 | -.01 |
| **Close to me** | 37 (18.3%) | -.01 | .03 |
| **n/a** | 123 (60.9%) | -.02 | -.08 |
| **Self-isolating due to government regulation or recommendation** |  |  |  |
| **Me** | 128 (63.4%) | .03 | .01 |
| **Close to me** | 88 (43.6%) | .12 | .01 |
| **n/a** | 58 (28.7%) | -.12 | -.07 |
| **Currently living alone** | 45 (20.6%) | .07 | -.09 |

* indicates *p* < .05. ** indicates *p* < .01. T3 – Data collection at Time 3, taking place from April 15-April 20, 2020.

Table 2. Equivalence testing of clinical measures and clinically relevant traits

| **Comparison** | **M (SD)** | **Cohen’s *d* [90% CI]** | **NHST test for differences** | **TOST test for equivalence** | **Conclusion** |
| --- | --- | --- | --- | --- | --- |
| **Depression** |  |  |  |  |  |
| **T1**  **T2** | 5.89 (5.48) 5.77 (5.62) | -0.02 [-0.11; 0.06] | *t*(201) = 0.52, *p* = .607 | *t*(201) = 2.77, *p* = .003 | Equivalent and not different |
| **T2**  **T3** | 5.77 (5.62) 6.38 (5.59) | 0.11 [0.00; 0.21] | *t*(201) = 2.05, *p* = .042 | *t*(201) = 5.34, *p* < .001 | Equivalent and different |
|  |  |  |  |  |  |
| **Anxiety** |  |  |  |  |  |
| **T1**  **T2** | 3.16 (3.95) 3.24 (3.82) | 0.02 [-0.08; 0.12] | *t*(201) = 0.42, *p* = .678 | *t*(201) = 3.71, *p* < .001 | Equivalent and not different |
| **T2**  **T3** | 3.24 (3.82) 2.86 (3.68) | -0.10 [-0.21; 0.01] | *t*(201) = 1.86, *p* = .065 | *t*(201) = 1.3, *p* = .097 | Not equivalent and not different |
|  |  |  |  |  |  |
| **Rumination** |  |  |  |  |  |
| **T1**  **T2** | 42.90 (11.07) 42.17 (11.86) | -0.06 [-0.15; 0.02] | *t*(201) = 1.46, *p* = .146 | *t*(201) = 1.94, *p* = .027 | Equivalent and not different |
| **T2**  **T3** | 42.17 (11.86) 41.05 (10.10) | -0.04 [-0.13; 0.05] | *t*(201) = 0.91, *p* = .366 | *t*(201) = 1.69, *p* = .046 | Equivalent and not different |
|  |  |  |  |  |  |
| **Distress Tolerance** |  | |  |  |  |
| **T1**  **T2** | 43.06 (10.23) 41.99 (9.93) | -0.11 [-0.21; 0.00] | *t*(201) = 1.98, *p* = .049 | *t*(201) = 1.24, *p* = .109 | Not equivalent and different |
| **T2**  **T3** | 41.99 (9.93) 41.04 (10.10) | -0.09 [-0.20; 0.01] | *t*(201) = 1.76, *p* = .080 | *t*(201) = 2.87, *p* = .002 | Equivalent and not different |

Note. T1 – Data collection at Time 1, October 17, 2018. T2 – Data collection at Time 2, April 15-22, 2019. T3 – Data collection at Time 3, April 15-20, 2020.

Table 3. Summary of model fit statistics for the alternative path models

| **Model** | **χ^2^**  **(*df*)** | ***p* value** | **Robust CFI** | **Robust RMSEA** | **RMSEA 90% CI** | **Robust SRMR** |
| --- | --- | --- | --- | --- | --- | --- |
| **Depression models** |  |  |  |  |  |  |
| **Loneliness -> depression model (Figure 3a)** | 25.80 (2) | <.001 | .000 | .241 | .163-.329 | .001 |
| **Depression -> loneliness model (Figure 4a)** | .290 (2) | .87 | 1.00 | 0.00 | 0.00-0.73 | .014 |
|  |  |  |  |  |  |  |
| **Anxiety models** |  |  |  |  |  |  |
| **Stress -> anxiety model (Figure 3b)** | 12.24 (2) | .002 | 0.00 | .160 | .082-.251 | .002 |
| **Anxiety -> stress model (Figure 4b)** | .80 (2) | .669 | 1.00 | .000 | .000-.107 | .030 |

CFI = comparative fit index; RMSEA = root-mean-square error of approximation; CI = confidence interval; SRMR = Standardized root mean square residual.

Figures 3a-b. Structural equation model where COVID-19 loneliness/stress predicts depression/anxiety beyond baseline. ^***^  *p* < .001, ^*^ *p* ≤ .05, † *p* < .10. T2 – Data collection at Time 2, April 15-20, 2019. T3 – Data collection at Time 3, April 15-20, 2020. The above models were bad fits for the data (e.g., CFI = .000) and were therefore rejected.

**
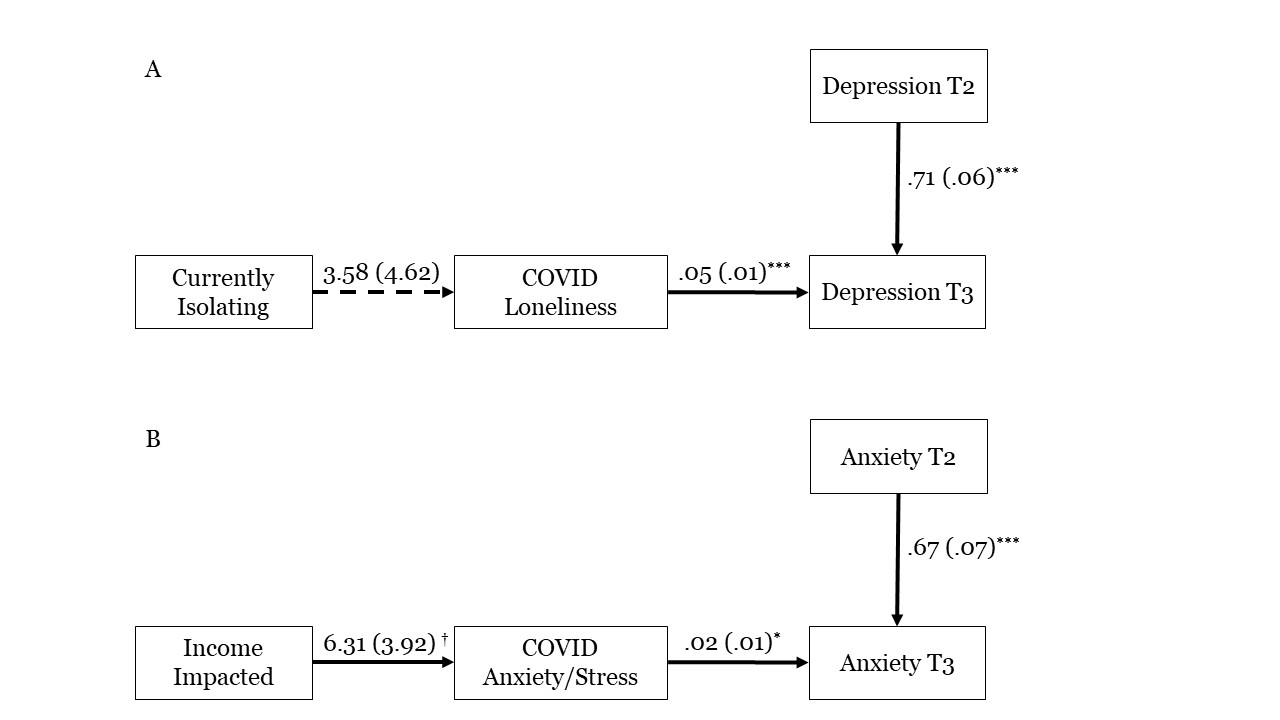
**

Figures 4a-b. Structural equation model where COVID-19 depression/anxiety predicts COVID-19 loneliness/stress. ^***^  *p* < .001, ^*^ *p* ≤ .05, † *p* < .10. T2 – Data collection at Time 2, April 15-20, 2019. T3 – Data collection at Time 3, April 15-20, 2020. The above models were excellent fits for the data (e.g., CFI = 1.00) and were therefore retained.


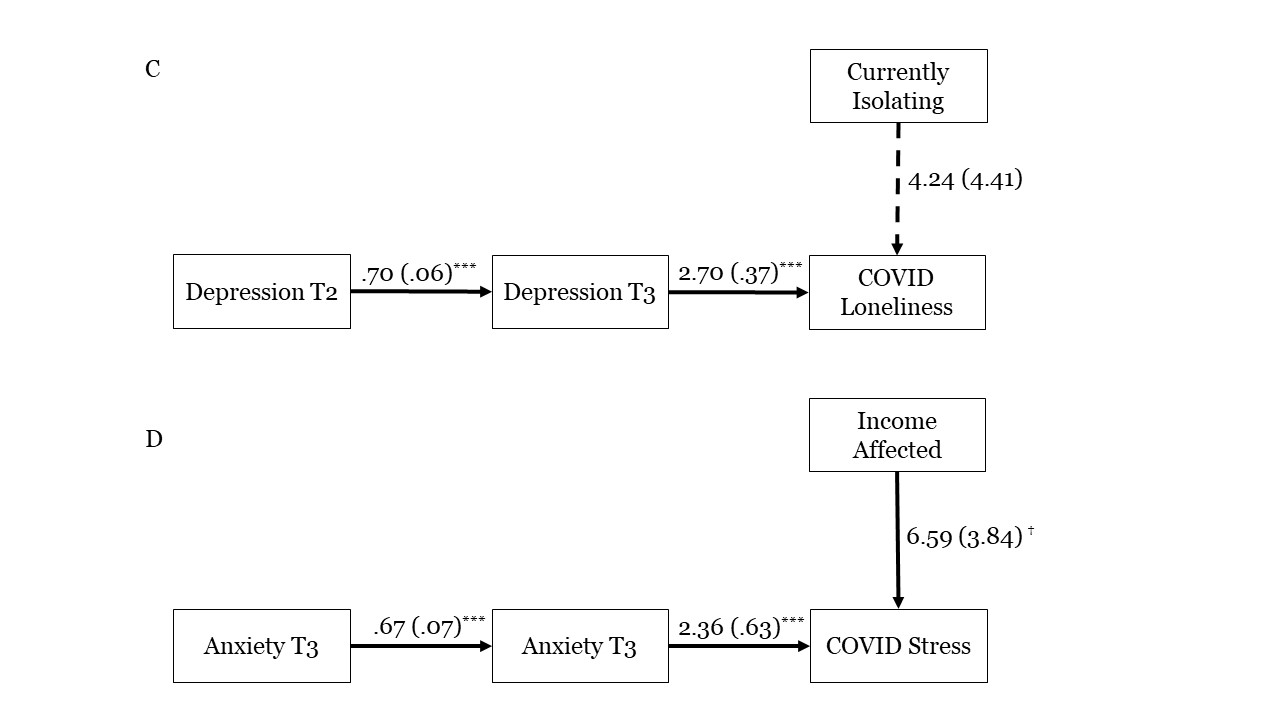

Supplement: S1 File — (DOCX) [file pone.0273945.s003.docx]
