# Supplementary material for: Mood symptoms predict COVID-19 pandemic distress but not vice versa: An 18-month longitudinal study
Source: PLoS One. 2022 Sep 2;17(9):e0273945. doi: 10.1371/journal.pone.0273945 (PMC9439223; doi:10.1371/journal.pone.0273945)
Supplement: Attachment — Submitted filename: PLOS One Review Mood Symptoms Predict COVID (KSB, 7-27-22).pdf [file pone.0273945.s007.pdf]

Mood Symptoms Predict COVID-19 Pandemic Distress but not Vice Versa:
An 18-Month Longitudinal Study

Benjamin A. Katz,[1]* Iftah Yovel[1]

KSB Review (7-27-22)

1st review comments (1-16-22): Yellow

2nd review comments (7-27-22): Blue

p. 3

"The high levels of health and financial uncertainly salient to the pandemic are strongly linked to internalizing pathology [3]."

What about "externalizing pathology". Seems important to reference as well.

My question re: externalizing pathology was not addressed by the authors. I would assume there are a lot of data demonstrating that the pandemic was correlated with increased marital/family conflict, increased drug use, increased aggression, etc. Even if the authors chose not to explore this in their article, I think it would be important to mention, briefly, as it reflects an oft discussed problem associated with social isolation.

p. 4

"Thus, in order to assess the COVID-19 pandemic's negative impact on mental health, it is necessary to identify participants with pre-pandemic baseline data available, and to separately assess clinical symptoms, environmental stressors, and subjective distress."

Thoughtful, complex design.

…

"The pandemic assessment period occurred between April 15, 2020 and April 20, 2020,.."

This research is limited by the lack of measurements 6 or 12 or 18 months into the pandemic, that reflects the effects of long-term, cumulative stress.

This was addressed by the authors. Perhaps I missed it the first time, or perhaps they added it, but I appreciate their recognizing the importance of longer-term followup.

p. 5-6

Where is the demographic data. It appears pre-disaster trauma history (e.g. ACES study) was not included. Given that interpersonal trauma could be a predictor of post-disaster/pandemic reactivity, this should at minimum be mentioned as a study limitation.

This has not been mentioned/addressed by the authors, and I think should at minimum be included in their discussion of the limitations of this study. On page 17, they wrote: "Thus, those most at risk for distress during the pandemic were those experiencing both environmental stressors relevant to the distress (e.g., economic stress) as well as a history of mental illness (e.g., anxiety)."

That is good as far as it goes, using baseline data, but ***it makes no reference to how trauma history might predict greater distress and psychopathology as response to the pandemic. I would still want this referenced, however briefly, around page 17.***

p. 6

"They represented a wide range of ages (M = 42.87, SD = 13.09, range = 19 – 75)"

It would be helpful to see the data on how many people in each age grouping, example 19-29, 30-39, etc. to see if age cohorts mattered or not.

The authors wrote: "Further demographic data is available in S1."

The authors did not explore whether there are any cohort differences wrt outcome. They also didn't explore whether there were significant outcome differences between white vs non-white, North America vs UK/Ireland. ***Again, I would like this at minimum referenced as a limitation worthy of further investigation.***

p. 9-10

"The relationships between the environmental stressors and symptom severity were assessed using ***polychoric correlations***. All other relationships (e.g., between subjective COVID-19 stress and symptom severity) were calculated using ***Pearson's correlations***."

I am not well-versed in statistics, which perhaps is why it would be helpful for the reader to understand why two different measurements of correlation were chosen, and what they each differentially show and don't show.

The authors now wrote: "The models included binary data (i.e., environmental stressors) and were therefore calculated using polychoric correlations and a diagonal weighted least squares (DWLS) estimator [27]."

As far as I can tell, this addressed my concern as stated above.

…

“After observing the unexpectedly small effect sizes of change between time-points, we next estimated whether these effects were statistically equivalent to zero. This was done using the two one-sided tests (TOST) procedure for equivalence testing [26].”

It is my understanding the more tests you run, the more you’re likely to “find something” of statistical significance. Is this taken into account? If so, specify how.

I’m not sure this was addressed by the authors, but I am not a statistician so perhaps it was considered when the authors wrote: “Models were assessed using robust fit statistics using the recommended cutoffs [28,29] of: non- significant chi-square test, CFI > .95, RMSEA < .06, SRMR < .08.”

p. 13

“Distress intolerance, on the other hand, was not found to be significantly equivalent (p = .096) across timepoints. As such, distress intolerance was judged to be ***different between T1 and T2, to a significant albeit trivial degree***.”

Is p= .096 considered significant?? Shouldn’t it be lower than .05? Perhaps this should be explained as to why this is statistically significant, however “trivial”.

The authors have not addressed my concern at all. How can something be “***not significantly equivalent***” and “***different… to a significant albeit trivial degree***.” I understand p= .096 fits with “not significantly equivalent”, but then how do the authors conclude T1 and T2 are “different to a significant albeit trivial degree”? “Not equivalent” is not the same as “different to a significant degree”. Where is the data to support that second statement? Am I missing something?

p. 17

“While unexpected, these findings were consistent with patterns observed following other disasters. Populations appear to be resilient to large-scale environmental stressors [9].”

How much is this a function of this being very early in the pandemic? Would it change after 1 year? After getting the vaccine, and then new variants causing a resurgence?

Since the authors did followup at 12 and 18 months, this concern was addressed.

Also, the subject demographics does not assessment of interpersonal trauma history, e.g, along the lines of the ACES studies. In my clinical experience, the trauma history pre-“disaster” has a significant effect on post-disaster coping. At the minimum, the fact that interpersonal trauma history was NOT assessed should be mentioned.

As I highlighted above, p. 5-6, no mention was made of pre-pandemic trauma history. In my clinical experience and that of my colleagues, there was an intersection between

complex, childhood trauma and response to the pandemic. Again, I would want that stated as a limitation of this study.

…

"…those experiencing both environmental stressors relevant to the distress (e.g., economic stress) as well as a history of mental illness (e.g., anxiety)."

Again, trauma history is not included. Some of the anxiety, depression, isolation, etc. could be a consequence of post-trauma sequelae. Again, this limitation of this study needs mentioning.

Again, history of mental illness is an important variable, but trauma history may be as or more important. Mention this is worthy of further study.

<u>p. 18</u>

"Although it occurred during peak rates of COVID-19 fatality (see Figure 1), other events during the pandemic may have led to other distress peaks as well, such as when participants began to self-isolate [33]."

This is a good point.

..

"Identifying which participants are most vulnerable to mental illness in the face of pandemic-related stress is the first step in developing targeted interventions [35]. In doing so, the clinical science community may rise to the formidable challenge that COVID-19 has set before it."

Agreed, but I don't think the researchers take into account what I believe (and I assume research has shown) the importance of interpersonal, particularly severe childhood trauma history (a la the ACES research) and neglect. If you don't take into account childhood trauma and neglect, your interventions will not be as patient-specific and effective as desired.

On page 18, the authors wrote: "Collaborative work between multiple laboratories will also offer opportunities to ***compare findings among diverse populations and assessment methods*** in order to more efficiently identify at-risk populations [34].

Not to beat a dead horse, but what I highlighted speaks directly to my concern that pre-pandemic, and especially early childhood, relational trauma history was not assessed. Since the issue of relational trauma addresses both matters of "***population***" and "***assessment***", this would be the place to mention trauma history as important in "further/future study."
