# Supplementary material for: Mood symptoms predict COVID-19 pandemic distress but not vice versa: An 18-month longitudinal study
Source: PLoS One. 2022 Sep 2;17(9):e0273945. doi: 10.1371/journal.pone.0273945 (PMC9439223; doi:10.1371/journal.pone.0273945)
Supplement: Attachment — Submitted filename: PLoS Response to Reviewers.docx [file pone.0273945.s004.docx]

Editor’s comments

*1. Please ensure that your manuscript meets PLOS ONE's style requirements, including those for file naming*

We have implemented the following changes consistent with PLOS ONE’s style book:

- Headings, table titles and figure titles were adjusted in terms of location and font size.
- Citations were changed to PLOS ONE style instead of APA style.
- Tables and figure titles were integrated into the text of the manuscript and Supplementary Table 1 was noted at the end.

*2. Please update your submission to use the PLOS LaTeX template*

In keeping with PLOS ONE policy, we uploaded a .docx version of the manuscript with and without changes tracked.

*3. Please include your full ethics statement in the ‘Methods’ section of your manuscript file. In your statement, please include the full name of the IRB or ethics committee who approved or waived your study, as well as whether or not you obtained informed written or verbal consent. If consent was waived for your study, please include this information in your statement as well.*

The following ethics statement is now included at the end of the Method section:

The study was conducted with the approval of the Ethics Committee of the Faculty of Social Sciences at the Hebrew University of Jerusalem under the study, "A path model for the connection between reinforcement sensitivity theory, emotion regulation, and psychopathology". All participants provided written consent prior to each wave of assessments.

Additionally, all figures were converted to .tif format and passed through the Preflight Analysis and Conversion Engine (PACE) digital diagnostic tool prior to upload. We have also carefully followed all file naming conventions as requested.
