# Supplementary material for: Mood symptoms predict COVID-19 pandemic distress but not vice versa: An 18-month longitudinal study
Source: PLoS One. 2022 Sep 2;17(9):e0273945. doi: 10.1371/journal.pone.0273945 (PMC9439223; doi:10.1371/journal.pone.0273945)
Supplement: Attachment — Submitted filename: PLOS One Review Mood Symptoms Predict COVID (Benau, 1-16-22).docx [file pone.0273945.s005.docx]

Mood Symptoms Predict COVID-19 Pandemic Distress but not Vice Versa:

An 18-Month Longitudinal Study

Benjamin A. Katz,^1^* Iftah Yovel^1^

**KSB Review (1-16-22)**

**p. 3**

“The high levels of health and financial uncertainly salient to the pandemic are strongly linked to internalizing pathology [3].”

What about “externalizeing pathology”. Seems important to reference as well.

**p. 4**

“Thus, in order to assess the COVID-19 pandemic’s negative impact on mental health, it is necessary to identify participants with pre-pandemic baseline data available, and to separately assess clinical symptoms, environmental stressors, and subjective distress.”

Thoughtful, complex design.

…

“The pandemic assessment period occurred between April 15, 2020 and April 20, 2020,..”

This research is limited by the lack of measurements 6 or 12 or 18 months into the pandemic, that reflects the effects of long-term, cumulative stress.

**p. 5-6**

Where is the demographic data. It appears pre-disaster trauma history (e.g. ACES study) was not included. Given that interpersonal trauma could be a predictor of post-disaster/pandemic reactivity, this should at minimum be mentioned as a study limitation.

**p. 6**

“They represented a wide range of ages (M = 42.87, SD = 13.09, range = 19 – 75)”

It would be helpful to see the data on how many people in each age grouping, example 19-29, 30-39, etc. to see if age cohorts mattered or not.

**p. 9-10**

“The relationships between the environmental stressors and symptom severity were assessed using ***polychoric correlations***. All other relationships (e.g., between subjective COVID-19 stress and symptom severity) were calculated using ***Pearson’s correlations***.”

I am not well-versed in statistics, which perhaps is why it would be helpful for the reader to understand why two different measurements of correlation were chosen, and what they each differentially show and don’t show.

…

“After observing the unexpectedly small effect sizes of change between time-points, we next estimated whether these effects were statistically equivalent to zero. This was done using the two one-sided tests (TOST) procedure for equivalence testing [26].”

It is my understanding the more tests you run, the more you’re likely to “find something” of statistical significance. Is this taken into account? If so, specify how.

**p. 13**

“Distress intolerance, on the other hand, was not found to be significantly equivalent (p = .096) across timepoints. As such, distress intolerance was judged to be different between T1 and T2, to a significant albeit trivial degree.”

Is p= .096 considered significant?? Shouldn’t it be lower than .05? Perhaps this should be explained as to why this is statistically significant, however “trivial”.

**p. 17**

“While unexpected, these findings were consistent with patterns observed following other disasters. Populations appear to be resilient to large-scale environmental stressors [9].”

How much is this a function of this being very early in the pandemic? Would it change after 1 year? After getting the vaccine, and then new variants causing a resurgence?

Also, the subject demographics does not assessment of interpersonal trauma history, e.g, along the lines of the ACES studies. In my clinical experience, the trauma history pre-“disaster” has a significant effect on post-disaster coping. At the minimum, the fact that interpersonal trauma history was NOT assessed should be mentioned.

…

“…those experiencing both environmental stressors relevant to the distress (e.g., economic stress) as well as a history of mental illness (e.g., anxiety).”

Again, trauma history is not included. Some of the anxiety, depression, isolation, etc. could be a consequence of post-trauma sequelae. Again, this limitation of thes tudy needs mentioning.

**p. 18**

“Although it occurred during peak rates of COVID-19 fatality (see Figure 1), other events during the pandemic may have led to other distress peaks as well, such as when participants began to self-isolate [33].”

This is a good point.

..

“Identifying which participants are most vulnerable to mental illness in the face of pandemic-related stress is the first step in developing targeted interventions [35]. In doing so, the clinical science community may rise to the formidable challenge that COVID-19 has set before it.”

Agreed, but I don’t think the researchers take into account what I believe (and I assume research has shown) the importance of interpersonal, particularly severe childhood trauma history (a la the ACES research) and neglect. If you don’t take into account childhood trauma and neglect, your interventions will not be as patient-specific and effective as desired.
