# Supplementary material for: Mood symptoms predict COVID-19 pandemic distress but not vice versa: An 18-month longitudinal study
Source: PLoS One. 2022 Sep 2;17(9):e0273945. doi: 10.1371/journal.pone.0273945 (PMC9439223; doi:10.1371/journal.pone.0273945)
Supplement: Attachment — Submitted filename: PLOS One Reviews.docx [file pone.0273945.s006.docx]

Reviewer #1:

p. 3

“The high levels of health and financial uncertainly salient to the pandemic are strongly linked to internalizing pathology [3].”

What about “externalizing pathology”. Seems important to reference as well.

*We have now included a citation for externalizing pathology in the sentence (p. 3):*

*The high levels of health and financial uncertainly salient to the pandemic are strongly linked to stress, [7] internalizing pathology [8] and externalizing pathology [9].*

p. 4

“The pandemic assessment period occurred between April 15, 2020 and April 20, 2020,..”

This research is limited by the lack of measurements 6 or 12 or 18 months into the pandemic, that reflects the effects of long-term, cumulative stress.
*We have now included this point in the limitation section (pp. 23):*

*Furthermore, the current research design focuses on the acute periods of stress that accompanied the beginning of the pandemic. However, as the pandemic continued, stress has accumulated. Future studies may assess the association between cumulative stress and mental illness [47].*

p. 5-6

Where are the demographic data. It appears pre-disaster trauma history (e.g. ACES study) was not included. Given that interpersonal trauma could be a predictor of post-disaster/pandemic reactivity, this should at minimum be mentioned as a study limitation.

*Demographic data, including gender, location, race/ethnicity, age, education, and employment status, are available in S1 Table (p. 29). We have checked prior to uploading that this table is available for review.*

*Additionally, we now include the lack of assessment of adverse childhood experiences and other interpersonal factors as a limitation on p. 23:*

*Finally, the current study approaches risk factors for mental illness from the perspectives of intrapersonal processing and of acute stress. However, many risk factors both preceding and during the COVID-19 pandemic are interpersonal in nature. Social stressors both from the participant’s past (e.g., adverse childhood events) [47] and during their experiences in quarantine (e.g., domestic violence) [48] are themselves likely risk factors for loneliness, and mood disorder [49]. Future studies may further examine the role of interpersonal factors in well-being at the onset of the pandemic [47].*

p. 6

“They represented a wide range of ages (M = 42.87, SD = 13.09, range = 19 – 75)”

It would be helpful to see the data on how many people in each age grouping, example 19-29, 30-39, etc. to see if age cohorts mattered or not.

*This breakdown of ages is now included in the demographics table, S1 Table.*


p. 9-10

“The relationships between the environmental stressors and symptom severity were assessed using polychoric correlations. All other relationships (e.g., between subjective COVID-19 stress and symptom severity) were calculated using Pearson’s correlations.”

I am not well-versed in statistics, which perhaps is why it would be helpful for the reader to understand why two different measurements of correlation were chosen, and what they each differentially show and don’t show.

*Response:*

*To clarify this issue, we have now rewritten the beginning of the analysis plan to explain the use of polychoric vs Pearson correlations as well as how to interpret them (p. 11):*

*Analytic strategies were selected based on the psychometric properties of the variables. Environmental stressors were measured with binary data and as such were summarized using frequency statistics (i.e., rate and percentage). The relationships between the environmental stressors and symptom severity were assessed using the recommended polychoric correlations [38]. Subjective measures of COVID-19 stress, rumination, distress intolerance, depression, and anxiety were measured using dimensional variables. As such, they were summarized using descriptive statistics and their interrelationships were calculated using Pearson’s correlations. Polychoric correlations and Pearson’s correlations were evaluated using the same significance cutoff of p < .05 and their effect sizes were considered comparable to each other..*

…

“After observing the unexpectedly small effect sizes of change between time-points, we next estimated whether these effects were statistically equivalent to zero. This was done using the two one-sided tests (TOST) procedure for equivalence testing [26].”

It is my understanding the more tests you run, the more you’re likely to “find something” of statistical significance. Is this taken into account? If so, specify how.

*Response:*

*We certainly agree with the reviewer’s concern regarding degrees of freedom that researchers take for themselves. In keeping with this concern, we follow the Open Science movement approach that recommends transparent reporting of which analyses were chosen* a priori *and which were secondary (e.g., Benning et al., 2019). In this case, we found the data to be more appropriate for a TOST procedure, which incorporates both null hypotheses significance testing (NHST) and TOST. Importantly, we did not perform other tests on these data germane to the current study, and have now included a citation when describing the study that may refer interested readers to the other tests that have been performed on this dataset in a separate study (p. 10):*

*Participants were recruited via the Prolific Academic Platform as part of an ongoing study on reinforcement sensitivity, emotion regulation, and affective psychopathology [35]*

*Source: Benning, S. D., Bachrach, R. L., Smith, E. A., Freeman, A. J., & Wright, A. G. (2019). The registration continuum in clinical science: A guide toward transparent practices. Journal of Abnormal Psychology, 128(6), 528.*

p. 13

“Distress intolerance, on the other hand, was not found to be significantly equivalent (p = .096) across timepoints. As such, distress intolerance was judged to be different between T1 and T2, to a significant albeit trivial degree.”

Is p= .096 considered significant?? Shouldn’t it be lower than .05? Perhaps this should be explained as to why this is statistically significant, however “trivial”.

*Response:*

*All differences in time-points were subjected to two rounds of testing. First, we used null-hypothesis significance testing (NHST) to evaluate difference at the two time points. Then, we used the two one-sided tests (TOST) approach to examine equivalence. A significant NHST indicates difference, while a significant TOST indicates equivalence. In this case, NHST of distress tolerance from T1 to T2 previously found significant difference (d = -.10, p = .049). Then, at the point in analysis the reviewer references, we observed a failed TOST (*p *= .096), indicating non-equivalence. We thus concluded that the values were different, based on NHST, and non-equivalent, based on TOST.*

*To clarify this point, we have now updated the text of that section of analysis to clarify this difference (p. 13):*

*However, distress intolerance – which was found to be significantly different from T1 to T2 using NHST (d = .10, p = .049) – was not found to be significantly equivalent using TOST (p = .096).*

p. 17

“While unexpected, these findings were consistent with patterns observed following other disasters. Populations appear to be resilient to large-scale environmental stressors [9].”

How much is this a function of this being very early in the pandemic? Would it change after 1 year? After getting the vaccine, and then new variants causing a resurgence?

Also, the subject demographics does not assessment of interpersonal trauma history, e.g, along the lines of the ACES studies. In my clinical experience, the trauma history pre-“disaster” has a significant effect on post-disaster coping. At the minimum, the fact that interpersonal trauma history was NOT assessed should be mentioned.
…

“…those experiencing both environmental stressors relevant to the distress (e.g., economic stress) as well as a history of mental illness (e.g., anxiety).”

Again, trauma history is not included. Some of the anxiety, depression, isolation, etc. could be a consequence of post-trauma sequelae. Again, this limitation of the study needs mentioning.

*Response:*

*We now include these as limitations to the study (p. 23):*

*Finally, the current study approaches risk factors for mental illness from the perspectives of intrapersonal processing and of acute stress. However, many risk factors both preceding and during the COVID-19 pandemic are interpersonal in nature. Social stressors both from the participant’s past (e.g., adverse childhood events) [47] and during their experiences in quarantine (e.g., domestic violence) [48] are themselves likely risk factors for loneliness, and mood disorder [49]. Future studies may further examine the role of interpersonal factors in well-being at the onset of the pandemic [47]. Furthermore, the current research design focuses on the acute periods of stress that accompanied the beginning of the pandemic. However, as the pandemic continued, stress has accumulated. Future studies may assess the association between cumulative stress and mental illness [47].*

p. 18

“Identifying which participants are most vulnerable to mental illness in the face of pandemic-related stress is the first step in developing targeted interventions [35]. In doing so, the clinical science community may rise to the formidable challenge that COVID-19 has set before it.”

Agreed, but I don’t think the researchers take into account what I believe (and I assume research has shown) the importance of interpersonal, particularly severe childhood trauma history (a la the ACES research) and neglect. If you don’t take into account childhood trauma and neglect, your interventions will not be as patient-specific and effective as desired.

*We now mention adverse childhood events and other interpersonal risk factors in the new limitation paragraph (see above response).*

Reviewer #2

1. The Introduction should include some systematic reviews reporting the evidence of mental health issues during COVID-19 pandemic to emphasize the importance to investigate mental health during COVID-19 pandemic. Please see the following suggestions.
Rajabimajd, N., Alimoradi, Z., & Griffiths, M. D. (2021). Impact of COVID-19-related fear and anxiety on job attributes: A systematic review. Asian Journal of Social Health and Behavior, 4, 51-55
Olashore, A. A., Akanni, O. O., Fela-Thomas, A. L., & Khutsafalo, K. (2021). The psychological impact of COVID-19 on health-care workers in African Countries: A systematic review. Asian Journal of Social Health and Behavior, 4, 85-97
Alimoradi, Z., Ohayon, M. M., Griffiths, M. D., Lin, C.-Y., & Pakpour, A. H. (2022). Fear of COVID-19 and its association with mental health related factors: A systematic review and meta-analysis. BJPsych Open, 8, e73.
Alimoradi, Z., Lin, C.-Y., Ullah, I., Griffiths, M. D., & Pakpour, A. H. (2022). Item response theory analysis of Fear of COVID-19 Scale (FCV-19S): A systematic review. Psychology Research and Behavior Management, 15, 581-596.
Alimoradi, Z., Gozal, D., Tsang, H. W. H., Lin, C.-Y., Broström, A., Ohayon, M. M., & Pakpour, A. H. (2022). Gender-specific estimates of sleep problems during the COVID-19 pandemic: Systematic review and meta-analysis. Journal of Sleep Research, 31(1), e13432.
Alimoradi, Z., Broström, A., Tsang, H. W. H., Griffiths, M. D., Haghayegh, S., Ohayon, M. M., Lin, C.-Y., Pakpour, A. H. (2021). Sleep problems during COVID-19 pandemic and its’ association to psychological distress: A systematic review and meta-analysis. EClinicalMedicine, 36, 100916.

*We have now included updated citations from this list and others, including:*

*Alimoradi Z, Gozal D, Tsang HWH, Lin C, Broström A, Ohayon MM, et al. Gender‐specific estimates of sleep problems during the COVID‐19 pandemic: Systematic review and meta‐analysis. Journal of Sleep Research. 2022;31. doi:10.1111/jsr.13432*

*Alimoradi Z, Ohayon MM, Griffiths MD, Lin C-Y, Pakpour AH. Fear of COVID-19 and its association with mental health-related factors: systematic review and meta-analysis. BJPsych Open. 2022;8: e73. doi:10.1192/bjo.2022.26*

*Avena NM, Simkus J, Lewandowski A, Gold MS, Potenza MN. Substance use disorders and behavioral addictions during the COVID-19 pandemic and COVID-19-related restrictions. Frontiers in Psychiatry. 2021;12. doi:10.3389/fpsyt.2021.653674*

*Berman NC, Fang A, Hoeppner SS, Reese H, Siev J, Timpano KR, et al. COVID-19 and obsessive-compulsive symptoms in a large multi-site college sample. Journal of Obsessive-Compulsive and Related Disorders. 2022; 100727. doi:10.1016/j.jocrd.2022.100727*

*Bourmistrova NW, Solomon T, Braude P, Strawbridge R, Carter B. Long-term effects of COVID-19 on mental health: A systematic review. Journal of Affective Disorders. 2022;299: 118–125. doi:10.1016/j.jad.2021.11.031*

*Hawes MT, Szenczy AK, Klein DN, Hajcak G, Nelson BD. Increases in depression and anxiety symptoms in adolescents and young adults during the COVID-19 pandemic. Psychological Medicine. 2021; 1–9. doi:10.1017/S0033291720005358*

*Robinson E, Sutin AR, Daly M, Jones A. A systematic review and meta-analysis of longitudinal cohort studies comparing mental health before versus during the COVID-19 pandemic in 2020. Journal of Affective Disorders. 2022;296: 567–576. doi:10.1016/j.jad.2021.09.098*

*Shanahan L, Steinhoff A, Bechtiger L, Murray AL, Nivette A, Hepp U, et al. Emotional distress in young adults during the COVID-19 pandemic: evidence of risk and resilience from a longitudinal cohort study. Psychological Medicine. 2022;52: 824–833. doi:10.1017/S003329172000241X*

*Viner R, Russell S, Saulle R, Croker H, Stansfield C, Packer J, et al. School closures during social lockdown and mental health, health behaviors, and well-being among children and Adolescents during the first COVID-19 wave. JAMA Pediatrics. 2022;176: 400. doi:10.1001/jamapediatrics.2021.5840*

2. The authors tested two theoretical modes (i.e., general-stressor model and diathesis-stressor model). However, they did not introduce the two models in the Introduction. They only briefly mention the general concepts of the two models. However, given that the study’s main focus is to examine and compare the two models, the authors should elaborate the information and descriptions of the two models. It would be much better if the authors also use the figures to explain the two models in the Introduction.

*We have now expanded upon the two models in the introduction to give each theoretical model more space to be explained (pp. 3-4).*

*Researchers and public health specialists raised concerns that this time of acute stress would bring about a global spike in mental illness [12]. In doing so, they implicitly argue in favor of a general-stressor model, pointing out that the many disruptions and challenges presented by the COVID-19 pandemic have led to greater levels of cumulative stress across the population (Fig 1a). This population-level stress would transdiagnostically increase levels of mental illness on a population level [13]. Indeed, infection with COVID-19 has indeed been found to negatively impact mental health [14,15] and pandemic-related stress has been found to be associated with elevated symptom severity [16,17]. However, recent large-scale studies have found less support for the general stressor model on a population level, even at the beginning of the pandemic, a period of great stress and uncertainty. [6,18,19] Indeed, populations tend to be quite resilient in the face of disaster-related stress (for review, see [20]). For example, in 2012, Hurricane Sandy-related stress only predicted elevated symptoms among children predisposed to symptom-relevant affect; those high in temperamental sadness in a prior assessment showed elevated levels of depressive symptoms while those high in temperamental fearfulness showed elevated levels of anxiety symptoms [21]. Similarly, the COVID-19 pandemic may serve as a trigger for those with vulnerabilities to specific disorders as opposed to as a population-level stressor [22].*

*Furthermore, the theorized relationship between pandemic-related distress and psychopathology may follow an opposite causal direction as that presumed in a general-stressor model. Loneliness caused by self-isolation may lead to greater levels of depression as hypothesized [10,23]. However, in a diathesis-stress model (See Fig 1b), the opposite causal direction is also possible [13]. In such a case, individuals with more severe depression are themselves more sensitive to distress [24]. Those with more severe baseline symptom severity may be more sensitive to the loneliness experienced during self-isolation, particularly at the beginning [18,25]. Thus, in such a scenario, the observed association between depression and pandemic-related loneliness would still exist. [16] However, it would not be because pandemic-related loneliness led to greater levels of depression. Rather, in such a model, those who entered the pandemic with higher levels of depression would feel greater levels of loneliness during lockdown.*

*We have also included additional citations to support each approach and explicitly traced how each part of the model would relate to each other. Additionally, we have taken the reviewer’s suggestion to include a figure that displays these relationships, as preparation for the analyses that will be performed below (Figures 1a-1b).*

3. The majority of the participants were recruited from the US and the UK. Also, the authors described more COVID-19 information for the two countries. Therefore, I think that it is necessarily to do a sensitivity analysis on the US and UK samples (i.e., removing the Canada and Ireland participants to examined the tested models).

*We have repeated analyses using only samples from the US and UK and the resultant findings were remarkably similar. Average scores on the metrics of interest closely overlapped. For example, in the full sample, participants’ levels of pandemic-related anxiety were* M *= 57.40,* SD *= 28.00 while in the only UK-US sample, it was* M *= 57.54,* SD *= 27.51. Effect sizes from the main analyses were extremely similar as well. In the full sample, for example, depression from T1 to T2 changed with a Cohen’s* d *of -.02 [-0.10; 0.06]. In the UK-US sample, Cohen’s* d *for this effect was -.02 [-0.11; 0.06]. We thus feel that the main findings were not driven by the inclusion of participants from Ireland and Canada. We now include the full re-analysis as a supplemental material (S1 File) for others who may have a similar concern.*

4. The description of DASS is unclear. From the description of “seven-item subscales”, I know that the authors used DASS-21 instead of DASS-42. However, the authors did not make it clear that they have used DASS-21. Moreover, the citation credit should give to Lovibond and Lovibond (1995), as they are the original developers. It is fine to cite Henry and Crawford. However, Lovibond and Lovibond cannot be excluded in the citations.

*We have updated the description to clarify the use of the DASS-21 and included the Lovibond & Lovibond citation as well.*

5. The authors should provide scoring information for all the instruments. Also, the meaning of the directions in each instrument should be provided. Without such information, one cannot interpret the scores for the instruments.

*We have provided information for each scale’s item scoring system, potential range, and meaning of higher scores.*

6. From the Procedure section, one can understand that the attrition rate of the longitudinal study was about 40%. This is fine. However, the authors should provide information regarding whether the retained participants and the lost-to-follow-up participants share similar demographics. This can check if the missing is at random.

*We have reviewed attrition rates and demographic information about the participants and found that those who dropped out were higher in depression and anxiety to a small degree. These differences in depression (M (SD) = 5.85 (5.64) vs 7.76 (5.97)) and anxiety (M (SD) = 3.25 (3.91) vs 4.33 (4.25)), however, were not judged to be substantial enough to establish a clinically significant difference between the groups. Furthermore, because participants were only included if they completed all three assessments, we were not concerned about this difference impacting sample scores across timepoints.*

*We do feel, however, that readers would want to be informed of this difference in order to better evaluate the results. As such, we have updated the manuscript in a number of ways. First, we now report these differences in the text of the Procedure section (p. 11):*

*Participants who returned for the final assessment showed small differences in symptom severity and moderate differences in age from those who did not (S1 File). Specifically, participants who returned were older than those who did not (M = 41.90, SD = 13.07 vs M = 34.07 SD = 10.28, d = .65, p < .001), slightly less depressed (M = 5.85, SD = 5.64 vs M = 7.76, SD = 5.97, d = .33, p = .004), and slightly less anxious (M = 3,25, SD = 3.91 vs M = 4.33, SD = 4.25, d = .27, p = .019). In order to minimize their effects on difference scores between timepoints, only participants who completed all three time-points were included in analyses.*

*Second, we include a table of differences in scores as a function of attrition in supplementary table S2 Table (p. 32).*

*Finally, we note this issue at the end of the Discussion (pp. 22-23):*

*Additionally, the differences between those who returned for the final assessment and those who did not is worthy of acknowledgement. Although the differences in symptom severity were small and of low clinical significance before the pandemic, it is possible that the participants who did not return for the final assessment were more at risk for symptom increase than those who did. In such a case, it is possible that had they been included, equivalence across timepoints may not have occurred. This hypothetical circumstance, however, is also inconsistent with the general stressor model that predicts symptom increase across the population. Furthermore, the current study is consistent with other large-scale studies that have also not found that symptom severity increased precipitously during the pandemic. [6,18,19] Further research may benefit from focusing on how the pandemic impacted well-being among participants at different levels of symptom severity.*

7. From the statement, “Participants were recruited via the Prolific Academic Platform as part of an ongoing study on reinforcement sensitivity, emotion regulation, and affective psychopathology”, I wonder whether the authors have some publications already published to make a citation here.

*We have now amended the text to read (p. 10) “Participants were recruited via the Prolific Academic Platform as part of an ongoing study on reinforcement sensitivity, emotion regulation, and affective psychopathology [35].”.*

8. Tables 2 and 3 are out of the size and cannot be read.

*We have* *adjusted the page orientation for these tables in order to include the full tables within the margins.*

9. For all the tables, the authors should use footnotes to provide definition of T1, T2, and T3.

*Tables and figures were updated to include definitions for each time-point (e.g., “*T1 – Data collection at Time 1, October 17, 2018. T2 – Data collection at Time 2, April 15-22, 2019. T3 – Data collection at Time 3, April 15-20, 2020.”)_
