# Supplementary material for: Mood symptoms predict COVID-19 pandemic distress but not vice versa: An 18-month longitudinal study
Source: PLoS One. 2022 Sep 2;17(9):e0273945. doi: 10.1371/journal.pone.0273945 (PMC9439223; doi:10.1371/journal.pone.0273945)
Supplement: Attachment — Submitted filename: PLOS One Reviews.docx [file pone.0273945.s008.docx]

Reviewer #1:

p. 3

“The high levels of health and financial uncertainly salient to the pandemic are strongly linked to internalizing pathology [3].”

My question re: externalizing pathology was not addressed by the authors. I would assume there are a lot of data demonstrating that the pandemic was correlated with increased marital/family conflict, increased drug use, increased aggression, etc. Even if the authors chose not to explore this in their article, I think it would be important to mention, briefly, as it reflects an oft discussed problem associated with social isolation.

*We have now expanded upon the role of externalizing pathology and recommended that the current findings should be considered against similar studies on externalizing disorders (p. 25):*

*Additionally, the current study operationalized mental health in terms of metrics of distress, loneliness, depression, and anxiety. However, other metrics of behavioral health, such as substance use and behavioral addictions, have also been associated with social isolation during the pandemic’s onset [9]. The current study may be considered against others related to externalizing disorders in order to assess trends across different types of psychopathology.*

p. 5-6

Where are the demographic data. It appears pre-disaster trauma history (e.g. ACES study) was not included. Given that interpersonal trauma could be a predictor of post-disaster/pandemic reactivity, this should at minimum be mentioned as a study limitation.

This has not been mentioned/addressed by the authors, and I think should at minimum be included in their discussion of the limitations of this study. On page 17, they wrote: “Thus, those most at risk for distress during the pandemic were those experiencing both environmental stressors relevant to the distress (e.g., economic stress) as well as a history of mental illness (e.g., anxiety).”

That is good as far as it goes, using baseline data, but it makes no reference to how trauma history might predict greater distress and psychopathology as response to the pandemic. I would still want this referenced, however briefly, around page 17.

*Following the paragraph mentioned by the reviewer now on pp. 21-22, we now include the importance of adverse childhood experiences and other interpersonal factors in the discussion and explicitly point to where other longitudinal studies may be used to assess such questions (p. 23):*

*Indeed, it also more generally highlights the importance of longitudinal data in identifying those most at risk for elevated levels of mental illness during periods of great distress. For example, individuals with adverse childhood experiences may also be at risk for increased levels of mental illness during times of pandemic-related stress [47]. In this case, ongoing, lifetime longitudinal studies are specially equipped to assess the relationship between diatheses related to earlier experiences and later distress [16].*

p. 6

“They represented a wide range of ages (M = 42.87, SD = 13.09, range = 19 – 75)”

The authors wrote: “Further demographic data is available in S1.”

The authors did not explore whether there are any cohort differences wrt outcome. They also didn’t explore whether there were significant outcome differences between white vs non-white, North America vs UK/Ireland. **Again, I would like this at minimum referenced as a limitation worthy of further investigation**.

*Owing to the limited sample size and distribution of participants across varied locations, we are concerned that applying the number of comparisons in this suggestion may yield false positives in terms of group differences in effect size. However, consistent with the reviewer’s request, we now reference the role of demographics on COVID-related depression and anxiety in the discussion, highlighting the role of syndemic interactions between minoritized identity and the effects of the pandemic. We agree with the reviewer that interactions such as these are worthy of future inquiry, and, consistent with the reviewer’s request, note this as a limitation in the current study’s analysis and highlight this point as being worthy of further investigation on p. 23:*

*These findings may complement others that use demographic correlates to identify those at higher risk for elevated mental illness during the pandemic. Factors such as age, gender, race, ethnicity, location, and education are associated with internalizing pathology at the onset of the pandemic, alongside pandemic-related stressors [44]. These different risk factors interact syndemically, wherein pandemic-related stressors have greater impacts upon mental health for those with minoritized racial identities [45] or live in lower-income communities [46]. While the current study’s sample size is too small to undertake such interaction analyses, other large-scale studies will be better equipped to do so.*

p. 13

“Distress intolerance, on the other hand, was not found to be significantly equivalent (p = .096) across timepoints. As such, distress intolerance was judged to be different between T1 and T2, to a significant albeit trivial degree.”

Is p= .096 considered significant?? Shouldn’t it be lower than .05? Perhaps this should be explained as to why this is statistically significant, however “trivial”.

The authors have not addressed my concern at all. How can something be “not significantly equivalent” and “different... to a significant albeit trivial degree.” I
understand p= .096 fits with “not significantly equivalent”, but then how do the authors
conclude T1 and T2 are “different to a significant albeit trivial degree”? “Not
equivalent” is not the same as “different to a significant degree”. Where is the data to
support that second statement? Am I missing something?

*Response:*

*The TOST approach entails three steps when assessing group difference-vs-equivalence. First, we assess difference using null-hypothesis significance testing (NHST). For distress intolerance, his was the first test which was significant (i.e., “significantly different from T1 to T2 using NHST (d = .10, p = .049)”). Next, we assessed equivalence using the two one-sided tests (TOST) approach, which led to a non-significant outcome (i.e., “was not found to be significantly equivalent using TOST (p = .096)”). Finally, we assessed the size of this difference using Cohen’s* d. *Consistent with convention, effect sizes up to* d *= .10 were considered trivially small. We understand that the reviewer was also previously concerned that this would be an excess of tests for a single effect size. However, we would like to emphasize that this three-step process is the gold-standard approach for equivalence testing. We have added clarifications to the Analysis Plan section of the manuscript so that the systematic nature of these steps would be more clear (p. 12):*

*Assessing the extent to which depression and anxiety changed or remained the same between time points was done in three steps. First, we performed conventional null hypothesis significance tests (NHST) to examine differences between time-points. Specifically, within-group t tests were used, with symptom and trait measures (e.g., DASS-Depression) entered as the repeated measures across consecutive time-points (i.e., T1-T2 or T2-T3). In this case, a significant finding would indicate that the repeated measures changed between time-points.*

*After observing the unexpectedly small effect sizes of change between time-points, we next estimated whether these effects were statistically equivalent to zero. This procedure is performed using a combination of the previous NHSTs and an additional two one-sided tests (TOST) procedure for equivalence testing [39]. In this procedure, an a priori smallest effect size of interest (SESOI) is calculated. Two one-sided t-tests then assess whether the full confidence interval of the change score estimate falls within the positive and negative SESOI (e.g., Cohen’s d = -.20 < X < .20). If so, the change score is judged as statistically equivalent to zero and scores are considered equivalent to each other. Finally, in the case of significant difference, we examined Cohen’s d of difference in order to assess the size of the difference between timepoints.*

p. 17

“…those experiencing both environmental stressors relevant to the distress (e.g., economic stress) as well as a history of mental illness (e.g., anxiety).”

Again, trauma history is not included. Some of the anxiety, depression, isolation, etc. could be a consequence of post-trauma sequelae. Again, this limitation of the study needs mentioning.

Again, history of mental illness is an important variable, but trauma history may be as
or more important. Mention this is worthy of further study.

…

those experiencing both environmental stressors relevant to the distress (e.g.,
economic stress) as well as a history of mental illness (e.g., anxiety).”

Again, trauma history is not included. Some of the anxiety, depression, isolation, etc.
could be a consequence of post-trauma sequelae. Again, this limitation of this study
needs mentioning.

Again, history of mental illness is an important variable, but trauma history may be as
or more important. Mention this is worthy of further study

*Response:*

*We have taken four steps to remedy this concern. First, we now mention in the Method section that childhood experiences were not assessed (p. 10):*

*They completed a series of self-report questionnaires related to reinforcement sensitivity, emotion regulation and affective pathology followed by an unrelated behavioral task (e.g., for similar procedure, see [36]). Questionnaires only assessed recent levels of psychopathology. Histories of psychopathology or childhood risk factors (e.g., adverse childhood events) were not assessed.*

*Second, in the discussion on p. 23, we now consider the role of lifetime longitudinal studies in identifying those who experienced ACEs (“Indeed, it also more…and later distress [16].” See response to p. 5-6 above for full quote).*

*Third, in the limitations section, we now expand upon the extant discussion of ACEs to highlight the importance of including measures of participants’ past experiences that occurred prior to the first baseline assessment (p. 24-25):*

*Finally, the current study approaches risk factors for mental illness from the perspectives of contemporary intrapersonal processing, acute stress, and internalizing psychopathology. However, many risk factors for elevated mental illness occurred prior to the original baseline measures included in the study. Elevated levels of psychopathology prior to assessment [24] or adverse childhood events [47] are both useful for identifying those at risk for greater levels of distress while facing pandemic-related stressors. Future studies using similar designs may use retrospective assessments to capture some of these risk factors as well.*

Finally, we now highlight the importance of assessing clinical history as a future direction of research in the closing paragraph (*p. 25)*:

*Collaborative work between multiple laboratories will also offer opportunities to compare findings among diverse populations, assessment methods, and clinical histories in order to more efficiently identify at-risk populations [50].*

p. 18

“Identifying which participants are most vulnerable to mental illness in the face of pandemic-related stress is the first step in developing targeted interventions [35]. In doing so, the clinical science community may rise to the formidable challenge that COVID-19 has set before it.”

Agreed, but I don’t think the researchers take into account what I believe (and I assume research has shown) the importance of interpersonal, particularly severe childhood trauma history (a la the ACES research) and neglect. If you don’t take into account childhood trauma and neglect, your interventions will not be as patient-specific and effective as desired.

On page 18, the authors wrote: “Collaborative work between multiple laboratories will
also offer opportunities to compare findings among diverse populations and assessment methods in order to more efficiently identify at-risk populations [34].

Not to beat a dead horse, but what I highlighted speaks directly to my concern that prepandemic, and especially early childhood, relational trauma history was not assessed. Since the issue of relational trauma addresses both matters of “population” and “assessment”, this would be the place to mention trauma history as important in
“further/future study.”

*Please see the response to p. 17 for our four changes to the manuscript that were made in order to more explicitly highlight the role of childhood in symptom change during the pandemic.*
